# Quality of life in the over-80-year-old medical patient following intensive care

**DOI:** 10.1186/cc9920

**Published:** 2011-03-11

**Authors:** KJ Rowe, M Trivedi, A Ercole, K Gunning

**Affiliations:** 1 Addenbrookes Hospital, Cambridge, UK

## Introduction

The aim of this study was to determine the quality of life (QOL) in patients over 80 years old following intensive care who were admitted with a medical diagnosis. The older ICU population is increasing, and using QOL after critical illness rather than mortality may represent a better outcome measure. The evidence is conflicting, with some studies suggesting good QOL scores in the older patients compared with younger patients, whilst others show the opposite. This may be due to differences in study design with variations in age group studied and the follow-up period. Our study uses a novel approach to evaluate QOL using aged-matched controls.

## Methods

A total of 296 patients aged ≥80 years with a medical diagnosis were admitted to the ICU between 1 January 2006 and 31 December 2009. Patients alive in May 2010 were sent two questionnaires, one assessing subjective changes before and after ICU admission in four key areas (QOL, physical ability, mood and memory) and a second validated QOL scoring tool (SF-36). Patient views regarding their ICU stay were also explored. A control group of age-matched patients was identified from outpatient clinics and given similar questionnaires to complete.

## Results

Of 261 ICU admissions fulfilling the study criteria, 201 survived to ICU discharge and 148 to hospital discharge (73.6%). Of these, 81 were alive in May 2010. Forty-nine were sent questionnaires and 27 were returned (55%). Questionnaires were sent to 33 controls. Questionnaire 1 (subjective QOL) - in all key areas patients felt that their QOL had decreased following admission to ICU. Questionnaire 2 (SF-36) - there was no statistical difference between patients and controls in any of the SF-36 domains (Mann-Whitney U test, *P *< 0.05). Views regarding intensive care: 24/25 former ICU patients believed that admission to intensive care was in their best interest, 21/25 would want to be treated in intensive care again if needed, compared with 15/27 of controls. Eleven out of 25 former ICU patients had discussed their wishes regarding future treatment on intensive care with someone compared with 6/28 controls.

## Conclusions

The SF-36 results indicated that QOL scores in elderly survivors of medical intensive care are not significantly below those of their peers. There is, however, a subjective reduction in QOL. The majority of ICU survivors in this age group would want such treatment again.
